# Single-molecule optical microscopy of protein dynamics and computational analysis of images to determine cell structure development in differentiating *Bacillus subtilis*

**DOI:** 10.1016/j.csbj.2020.06.005

**Published:** 2020-06-09

**Authors:** Adam J.M. Wollman, Katarína Muchová, Zuzana Chromiková, Anthony J. Wilkinson, Imrich Barák, Mark C. Leake

**Affiliations:** a Departments of Physics and Biology, University of York, York YO10 5DD, United Kingdom; b Institute of Molecular Biology, Slovak Academy of Sciences, Bratislava, Slovakia; c Structural Biology Laboratory, Department of Chemistry, University of York, York YO10 5DD, United Kingdom

**Keywords:** Single-molecule, Sporulation, Super-resolution, Morphogenesis, Differentiation

## Abstract

Here we use singe-molecule optical proteomics and computational analysis of live cell bacterial images, using millisecond super-resolved tracking and quantification of fluorescently labelled protein SpoIIE in single live *Bacillus subtilis* bacteria to understand its crucial role in cell development. Asymmetric cell division during sporulation in *Bacillus subtilis* presents a model system for studying cell development. SpoIIE is a key integral membrane protein phosphatase that couples morphological development to differential gene expression. However, the basic mechanisms behind its operation remain unclear due to limitations of traditional tools and technologies. We instead used advanced single-molecule imaging of fluorescently tagged SpoIIE in real time on living cells to reveal vital changes to the patterns of expression, localization, mobility and stoichiometry as cells undergo asymmetric cell division then engulfment of the smaller forespore by the larger mother cell. We find, unexpectedly, that SpoIIE forms tetramers capable of cell- and stage-dependent clustering, its copy number rising to ~ 700 molecules as sporulation progresses. We observed that slow moving SpoIIE clusters initially located at septa are released as mobile clusters at the forespore pole as phosphatase activity is manifested and compartment-specific RNA polymerase sigma factor, σ^F^, becomes active. Our findings reveal that information captured in its quaternary organization enables one protein to perform multiple functions, extending an important paradigm for regulatory proteins in cells. Our findings more generally demonstrate the utility of rapid live cell single-molecule optical proteomics for enabling mechanistic insight into the complex processes of cell development during the cell cycle.

## Graphical abstract

**Figure:**
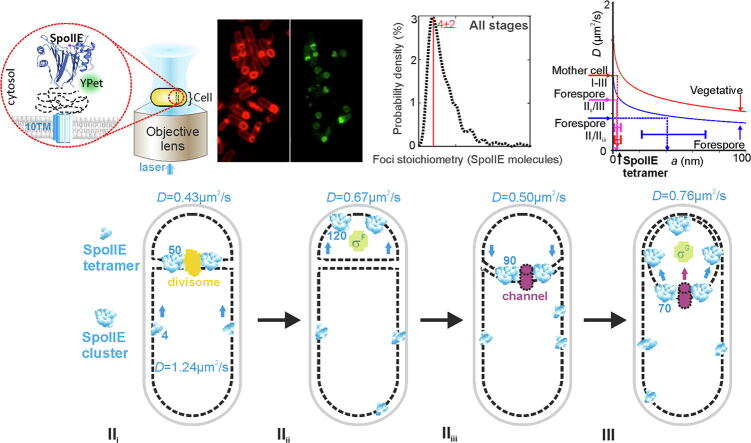

## 1 Introduction

**Fig. 1:**
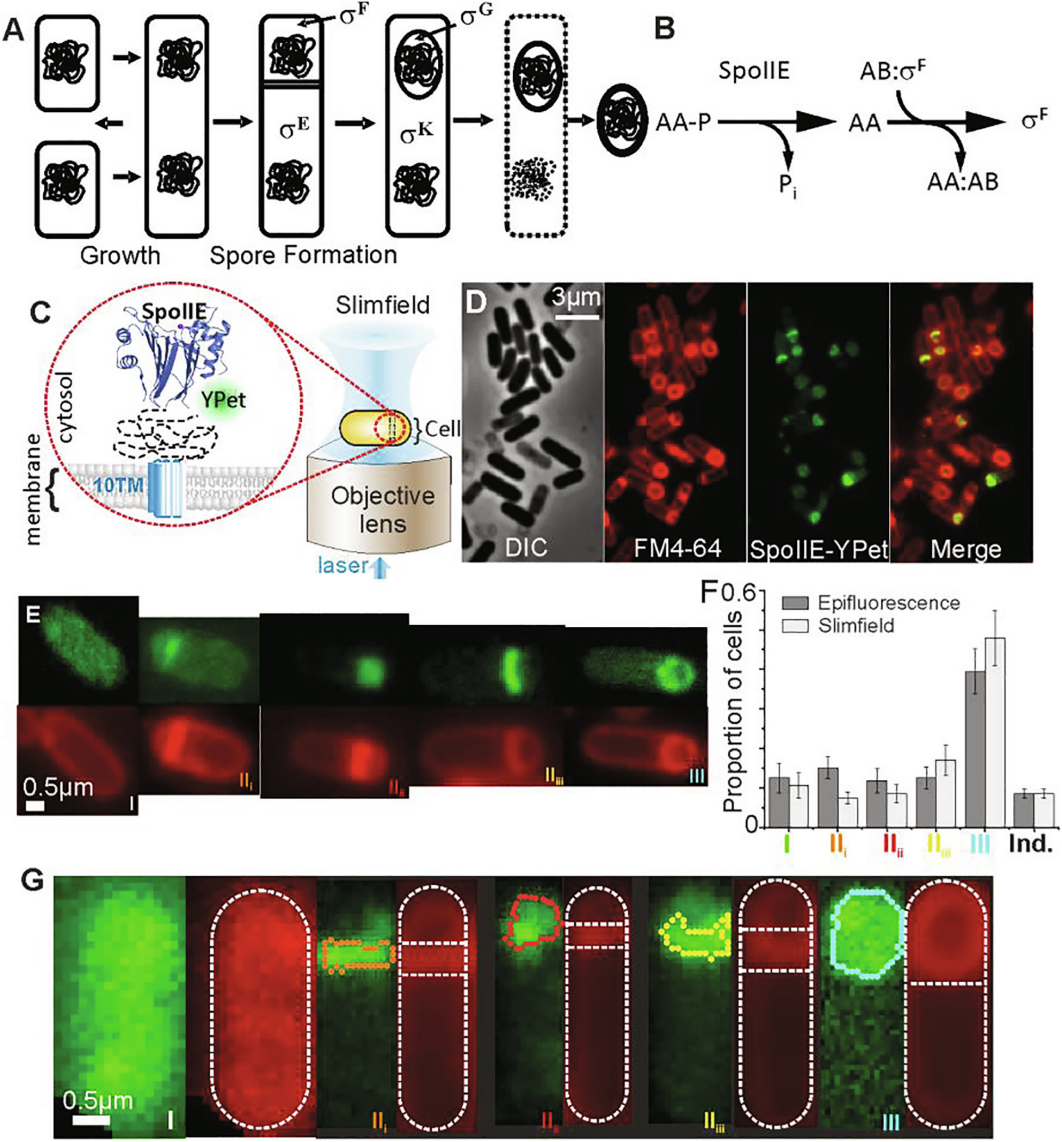
. Fluorescence imaging and classification by sporulation stage (A) Growth and sporulation of *B. subitlis*. During spore formation, the cell divides asymmetrically producing a smaller forespore and a larger mother cell. Compartment and stage specific sigma factors are activated sequentially. The forespore is engulfed by the mother cell before maturing into a resistant spore which is released when the mother cell lyses. (B) The SpoIIE phosphatase is the most upstream-acting of three proteins regulating the activity of the first compartment-specific sigma factor, σ^F^. Dephosphorylation allows SpoIIAA (AA) to displace σ^F^ from its complex with an anti-sigma factor (AB) enabling forespore-specific gene expression to be established. (C) Slimfield schematic, inset showing septum-localised SpoIIE-mYPet. (D) phase/epifluorescence microscopy of SpoIIE at different stages. Membrane labelling, FM4-64 (red), SpoIIE-mYPet (green). (E) Example epifluorescence images of SpoIIE-mYPet (green) with membraned stained with FM4-64 (red) at each stage. (F) Detected mean proportion of each stage or indeterminate (Ind.) (SEM error) of N = 4 fields of view each containing approximately N = 100 cells. Proportions were statistically identical between epifluorescence and slimfield data at p = 0.05 (p = 0.40,0.08, 0.29, 0.72, 0.72,0.49 for each stage/indeterminate respectively) (G) Categorization of stages from Slimfield, detected forespore/septa features (coloured lines), cell boundary segmentation ([33], [44]) based on fitting a sausage shape to fluorescence image indicated (outer white dash) and interface between forespore, septa and mother cell (horizontal white dash). (For interpretation of the references to color in this figure legend, the reader is referred to the web version of this article.)

Spore formation in *B. subtilis* offers a model system for studying development, differentiation, morphogenesis, gene expression and intercellular signalling in complex organisms [1], [2]. In nutrient rich conditions, rod-shaped cells grow and multiply by symmetric mid-cell division to generate identical daughters (Fig. 1A). However, when starved, *B. subtilis* ceases growth and is able to embark on a pathway of differentiation to form a dormant cell called a spore. Spore formation begins with an asymmetric division producing a smaller forespore cell next to a larger mother cell. Each compartment inherits an identical chromosome, but the patterns of gene expression, orchestrated by compartment-specific RNA polymerase sigma factors, differ resulting in alternative cell fates. The mother cell engulfs the forespore in a phagocytosis-like process creating a cell-within-a-cell (Fig. 1A), and a nurturing environment in which a robust multi-layered coat is assembled around the maturing spore [3]. In the final stages, the mother cell undergoes programmed cell death releasing the spore, which is resistant to multiple environmental stresses and can lie dormant until favourable growth conditions are restored.

At sporulation onset, ring-like structures of the tubulin homologue FtsZ form at mid-cell and migrate on diverging spiral trajectories towards the cell poles [4], colocalizing with the membrane integrated phosphatase PP2C SpoIIE [5]. One polar ring matures into the sporulation septum while the other disassembles [6]. Asymmetric division otherwise involves the same proteins as vegetative cell division, though the resulting sporulation septum is thinner [7], [8]. SpoIIE is the only sporulation-specific protein whose mutation causes ultrastructural changes in the asymmetric septum; null mutants of *spoIIE* are defective in sporulation and at lower frequency give rise to thicker asymmetric septa resembling the vegetative septum [7].

Changes in cell morphology during sporulation are coupled to a programme of gene expression, involving intercellular signalling, and the sequential activation of RNA polymerase sigma factors, σ^F^ and σ^G^ in the forespore and σ^E^ and σ^K^ in the mother cell [9]. Forespore-specific activation of σ^F^ on completion of the asymmetric septum is the defining step in differentiation. In pre-divisional and mother cells, σ^F^ resides in complex with the anti-sigma factor SpoIIAB while a third protein SpoIIAA is phosphorylated. After septation, SpoIIAA ~ P is dephosphorylated by the manganese-dependent protein phosphatase SpoIIE. The resulting SpoIIAA displaces σ^F^ from the σ^F^:SpoIIAB complex allowing RNA polymerase binding and transcription of forespore-specific genes (Fig. 1B) [10], [11]. This in turn, triggers activation of σ^E^ in the mother cell and establishes alternate programmes of gene expression which determine different cell fates (Fig. 1A).

SpoIIE has multiple roles at different sporulation stages (Fig. 1A,B). Assembly of SpoIIE to form polar rings – “E-rings”, dependent on interaction with FtsZ [12], occurs during stage I, defined by the formation of an axial filament spanning the cell length and comprising two copies of the chromosome each tethered through its origin region to opposing cell poles. Formation of the asymmetric septum is defined as stage II_i_, during which SpoIIE interacts with the divisome components RodZ [13] and DivIVA [14]. After closure of the sporulation septum, the FtsZ ring disassembles. SpoIIE-mediated activation of σ^F^ correlates with release of SpoIIE from the sporulation septum, marking stage II_ii_ [13]. During stage II_iii_, SpoIIE interacts with SpoIIQ [15] the forespore component of an intercellular channel [16], [17], [18], crucial for later activation of σ^G^. Stage III is characterized by mother cell engulfment of the forespore; SpoIIE localizes around the forespore, but there are no data to suggest a specific role of SpoIIE in this or later stages [15].

An increased concentration of SpoIIE in the forespore relative to that in the mother cell has been proposed to account for the selective activation of σ^F^ in the emerging forespore. This may occur through equipartitioning SpoIIE into the mother cell and forespore septal membranes leading to a higher SpoIIE effective concentration in the forespore as a result of its ~ 6 fold smaller volume [19]. It has also been shown that there is selective proteolysis of SpoIIE in the mother cell through the action of the membrane bound ATP-dependent protease, FtsH [20]. Here, it is proposed selective oligomerization at the forespore pole, protects SpoIIE from proteolysis in this compartment and further increases the concentration difference between the cell compartments.

To explore the complex function of SpoIIE further, we sought to determine its dynamic molecular architecture in differentiating cells. We employ a rapid single-molecule optical proteomics technique [65] Slimfield imaging [21], [22], [23] capable of tracking single fluorescently-labelled SpoIIE molecules with millisecond sampling in live *B. subtilis* cells to super-resolved spatial precision. By using step-wise photobleaching of the fluorescent protein tags [24] we determine the stoichiometry of each tracked SpoIIE complex and quantify the precise number of SpoIIE molecules in the mother cell and forespore in each individual cell. Also, by analysing the mobility of SpoIIE foci via their mean square displacement with respect to time, we calculate the microscopic diffusion coefficient *D*, model this to determine the effective diameter of SpoIIE complexes and correlate these data with measured SpoIIE content. Importantly, our copy number estimates indicate that there are similar numbers of SpoIIE molecules in both the mother cell and the forespore compartments when the asymmetric septum forms: since the volume in the forespore is significantly smaller than that of the mother cell this finding reveals an order of magnitude higher SpoIIE concentration in the forespore, correlated to the increased activity of σ^F^. We find that the stoichiometry and diffusion of tracked SpoIIE is dependent on its interaction partners and morphological changes, suggesting its roles in sporulation are influenced by oligomeric composition and mobility. Interestingly, we detect higher order mobile, oligomeric SpoIIE, towards the cell pole, at the stage of sporulation when σ^F^ becomes selectively activated in the forespore, as previously proposed [20].

## 2 Methods

### 2.1 Strains and plasmids

Gene cloning in *B. subtilis*, unless specified, was performed using standard protocols [25] (Table S1). To construct pSGIIE-mGFP, used in the FRAP experiments, we used previously prepared pSGIIE-YPet [13]. A PCR fragment containing mGFP was prepared using mGFPKpnF:

5′ ATCATCATCGGTACCATGAGTAAAGGAGAAGAACTTTTCACTGGAGTTGTC 3′

and mGFPBamR2:

5′ atcatcatcggatccTTATTTGTATAGTTCATCCATGCCATGTG 3′

primers and a plasmid derivative pSG1729 containing *m*g*fp* as template [26]. To yield pSG-mGFP this fragment was KpnI/BamHI digested and cloned into a similarly cut pSGIIE-YPet. Subsequently a 360 bp KpnI fragment containing *spoIIE* C-terminus (obtained from KpnI/BamHI cut pSGIIE-YPet) was cloned into pSG-mGFP digested with KpnI to yield pSGIIE-mGFP.

*B. subtilis* liquid cultures were grown in DSM [25] supplemented with chloramphenicol (5 µg ml^−1^), erythromycin (1 µg ml^−1^) and lincomycin (25 µg ml^−1^) as required. Samples for microscopy on 1% agarose slides were taken 2 h after sporulation onset (from our measurements this would ensure that the majority of cells after the onset of sporulation would have reached the start of stage II). For membrane visualization, FM 4–64 (Molecular Probes) was used (0.2–1 μg ml^−1^). When necessary, cells were concentrated by centrifugation (3 min, 2,300 × g) and resuspended in a small volume of supernatant. Images and analysis were obtained with an Olympus BX63 microscope (Hamamatsu Orca-R^2^ camera) and Olympus CellP or Olympus Image-Pro Plus 6.0 software. Imaging was performed at room temperature.

As N-terminal, cytosolic tail of SpoIIE (residues 11 to 37) is responsible for its proteolysis by FtsH (20), it is not possible to determine by western blot if the protein was degraded due to the fluorescent tag (Fig. S1C). It is also impossible to select only early stage sporulating cells corresponding to our microscopy data in western analysis as the fusion protein construct SpoIIE-mYPet localizes to the membrane and the cells sporulate at the level of the wild type cells, we believe the fusion protein is expressed, is functional and is degraded as the untagged version. Also, we did not detect any cytoplasmic fluorescence consistent with cleaved fluorescent protein alone (background fluorescence was consistent with out of plane foci – see later section). Epifluorescence images showed integration into the membrane (Fig. S1A) and simulated images of membrane integrated SpoIIE were qualitatively the same as our Slimfield images (Fig. S1B).

### 2.2 Single-molecule optical proteomics

A dual-color bespoke single-molecule microscope was used as described previously [23], [27] which utilized narrow epifluorescence excitation [67], [76] of 10 μm full width at half maximum in the sample plane from a 514 nm 20mW laser (Obis LS, Coherent). The laser was propagated through a ~ 3x Keplerian beam de-expander. Illumination was directed onto an *xyz* nanostage (Mad City Labs, the Dane County, Wisconsin, USA), and emissions directed through a color splitter utilizing a dichroic mirror centered on 560 nm wavelength and emission 25 nm bandwidth filters centered at 542/594 nm (Chroma Technology Corp., Rockingham, Vermont, USA) onto an Andor iXon 128 emCCD camera, 80 nm/pixel. Brightfield imaging was performed with no gain (100 ms/frame), single-molecule imaging at maximum gain (5 ms/frame).

Foci were automatically detected using MATLAB (Mathworks) software enabling a spatial localization precision of 40 nm using iterative Gaussian masking, and automated *D* and stoichiometry calculation [49], [77]. The copy number in the mother cell or forespore was determined by summing pixel intensities within the compartment, correcting for low background autofluorescence measured from FM4-64 labeled wild type *B. subtilis*, then dividing by the characteristic SpoIIE-mYPet intensity [27]. The intensity of each foci was defined as the summed intensity inside a 5 pixel radius circle corrected for the local background, defined as the mean intensity in a 17 pixel square outside the circle [24]. If the signal to noise ratio of the foci, defined as the mean intensity divided by the standard deviation of the local background, was >0.4 it was linked into an existing track if within 5 pixels [31], [66], approximately matching the diffraction-limited point spread function width. Only tracks with 4 or more points were analyzed, a commonly used criterion by us and others in the single-particle tracking field [28], [29]. The characteristic SpoIIE-mYPet intensity was calculated from foci intensities found towards the end of the photobleach, confirmed to be single molecule from detection of single step-wise photobleach events in individual over-tracked (i.e. tracked beyond photobleaching), Chung-Kennedy [30], [68], [69] filtered (an edge preserving smoothing algorithm) SpoIIE-mYPet tracks (Fig. S2). The stoichiometry of tracked foci was determined by fitting the first 4 intensity values of each track with exponential:

$$I=I_{0}exp\left(-\frac{t}{t_{b}}\right)$$

*I* = foci intensity, *I _0_* = initial intensity, *t* = time since laser illuminated cell, *t_b_* = bleach time (determined by an exponential fit to all population foci intensity to be ~ 100 ms). *I_o_* was divided by the mYPet characteristic intensity to give the stoichiometry. Although sub-optimal for low stoichiometry foci, e.g. < 6 molecules per focus, this exponential method is effective over a broad range of stoichiometries [31].

The 2D mean square displacement (MSD) was calculated from a fitted foci centroid (*x*(*t*),y(*t*)) assuming a track of *N* consecutive frames, and a time interval τ = *n*Δ*t*, where *n* is a positive integer and Δ*t* the frame integration time [32]:

$$MSD\left(\tau \right)=MSD\left(n\Delta t\right)=\frac{1}{N-1-n}\sum_{i=1}^{N-1-n}\left\{{\left[x\left(i\Delta t+n\Delta t\right)-x\left(i\Delta t\right)\right]}^{2}+\left[{\left[y\left(i\Delta t+n\Delta t\right)-y\left(i\Delta t\right)\right]}^{2}\right]\right\}=4D\tau +4\sigma^{2}$$

The localization precision from tracking is given by *σ*, which we measure as 40 nm. *D* is estimated from a linear fit to the first three data points in the MSD *vs*. τ relation (i.e. 1 ≤ *n* ≤ 3) for each accepted track, with the fit constrained to pass through a point 4*σ*^2^ on the vertical axis corresponding to τ = 0, allowing *σ* to vary in the range 20 – 60 nm in line with the experimental range.

### 2.3 Frap

FRAP was carried out on a Zeiss LSM 510 Meta confocal system with Axiovert inverted microscope, fitted with Plan Apochromat 100x /1.4NA oil objective and temperature-controlled stage. A 488 nm wavelength laser excited GFP, emissions collected via a 498–564 nm bandpass filter. The strength of photobleaching in the region of interest was set to 10–20 iterations of 100 ms each to ensure maximal photobleaching of GFP inside and minimum photobleaching beyond.

### 2.4 Categorization of cell cycle stage

To determine the cell cycle stage during the sporulation process, the following algorithm was used:

1. Cell images were initially coarsely over-segmented by thresholding the brightfield image and then using an initial ellipse shape approximation to define the cell length [27]. We then manually optimised the cell width of a sausage function (rectangle capped with two hemicircles) that enclosed the mYPet fluorescence intensity in each cell above the level of background noise.
2. Cells were then cropped out of the original image using a bounding rectangle around the segmentation and automatically rotated parallel to the horizontal axis.
3. A more precise segmentation stage then followed. This consisted of a double threshold Otsu’s method, applied to a 5 frame average of the mYPet fluorescence image. Pixels whose intensity values were above the 2nd threshold and multiplied by the segmentation contain the spore feature – either the whole forespore or septa.
4. These pixel areas were split into distinct connected components or candidate spore features and their centroids and areas calculated automatically using standard MATLAB functions.
5. A region was accepted as the mYPet spore feature mask if:
  1. Its centroid is within 40% of either end of the cell.
  2. Its centroid is within ± 40% of the middle of the cell width.
  3. The area of its centroid was > 10 pixels (there was no upper threshold).
  4. It had the highest summed pixel intensity of all the regions.
6. If nothing was accepted, steps 5.1–5.4 were repeated once with the previously found regions excluded.
7. If nothing was still found then the cell is ‘pre-sporulation/stage I’.
8. The FM4-64 frame average was similarly segmented but the mask multiplied by the forespore mask to give the FM spore feature.
9. Both FM and mYPet spore feature Major/Minor Axis, Area and Orientation were calculated by fitting the shape to an ellipse function.
10. Both were then assigned into 2 shape categories based on the aspect ratio, > 1.2 – ‘septa’, otherwise ‘filled’ structure. These correspond to fluorescence only at the linearly extended septa or distributed about the forespore in a rounder shape.
11. If the FM segmentation was ‘septa’, the segmentation was morphologically ‘thinned’ and its linear curvature calculated.
12. Stages were then assigned as follows:

Stage I/pre-sporulation: no mYPet spore feature detected.

Stage II_i_: ‘septa’ FM and mYPet spore features with curvature < 1.

Stage II_ii_: ‘septa’ FM and ‘filled’ mYPet spore features.

Stage II_iii_: ‘septa’ FM and mYPet spore features with curvature > 1.

Stage III: ‘filled’ FM and mYPet spore features.

To confirm the spore categorization algorithm we tested it on a series of simulated images (Fig S1C). These were generated by integrating a model point spread function (PSF) over a 3D model for the cell and forespore shape and subsequently noising the image with Poisson noise based on real noise characteristics of our microscope [33]. The cell membrane was modelled as a hollow cylinder, capped with hemisphere shells at either end with 1 pixel thick walls. Stage II_i_ septa were modelled as cell width disks while stage II_iii_ septa were modelled as hemispherical shells. Released SpoIIE in stage II_ii_ was modelled as a hemispherical shell capped by a disk while in stage III, it was modelled as a spherical shell. The relevant features for 100 cells in each stage were simulated in the ‘mYPet’ and ‘FM4-64′ channels and run through the categorization algorithm as if they were real data with no noise, average noise and the most extreme noise observed in the data. Without noise. 100% of cells were correctly identified, dropping to at worst in stage II_ii_ 79% with average noise and in the extreme case, as low as 42%.

We attempted further confirmation using Principal Component Analysis (PCA), an approach typically used to identify specific conformations or orientations in cryo-electron microscopy data. Data, images in this case, can be broken down into a basis set of eigenvectors or eigenimages which when summed in proportion to their eigenvalues, recreate the original dataset. Its use in live cell fluorescence data is challenging due to the high heterogeneity in size, shape and intensity of the images. Thus spore images were all cropped to 16x16 pixels, rotated and aligned and their intensity normalised (Fig. S1H) before a basis set of eigenvectors were calculated by Hotelling’s deflation [34]. The distribution of eigenvalues was strongly biased towards the 1st eigenvector (Fig. S1I) however 3D scatter plots of the first 3 eigenvalues did show separation of the data, further confirming our categorization algorithm but not allowing us to categorise spores based on PCA alone.

### 2.5 Determining the contribution from out-of-focus SpoIIE-mYPet foci

To quantify the contribution from out-of-focus SpoIIE-mYPet foci (i.e. those not detected during tracking) into the membrane ‘pool’ (i.e. spatially extended membranous regions of fluorescence intensity not detected as distinct foci), we assumed that the number and stoichiometry of detected foci from within the depth of field were the same as those without and were uniformly distributed. Assuming a depth of field of ~ 350 nm, on the basis of expectations from the numerical aperture of the objective lens and peak emission wavelength, a mean cell width of ~ 0.9 µm (61) and that the focal plane is exactly on the cell midplane we estimate ~ 1/4 of the cell membrane lies in the depth of field of the microscope. Thus, to generate indicative estimates for copy number values per cell we extrapolated the total number of summed SpoIIE-mYPet in foci by a factor of 4x. For the stage II mother cell (Table S2), the mean total number of molecules in foci per cell is ~ 32 (Mean foci stoichiometry multiplied by mean number of foci per cell) which multiplied by 4 agrees with the mean copy number of 82 ± 42 to within experimental error. Using the same method on other stages either agrees or over or underestimates implying that there is no measurable ‘pool’ of SpoIIE i.e. all of the SpoIIE-mYPet fluorescence can be accounted for by foci.

### 2.6 Simulating the effects of different oligomeric states for SpoIIE on the predicted stoichiometry distribution from Slimfield analysis

To simulate the effects of different oligomeric states of SpoIIE-mYPet on the observed stoichiometry distribution from Slimfield image data we calculated the probability of foci overlap[35] in each individual cell using the number of detected foci and the area of the spore feature in that particular cell. This probability was used to generate the distribution of overlaps using a Poisson distribution, based on a stage specific frequency of overlap, *λ*. The predicted apparent stoichiometry distribution was then generated by convolving the overlap distribution with the intensity distribution of model stoichiometry, *S* (i.e. *S* = 2, dimers, *S* = 4, tetramers etc.). This intensity distribution was generated from the mYPet characteristic intensity distribution (Fig. S2C), re-centred on 2*S*, width scaled to *S*^1/2^*σ, where σ = 0.675, the sigma width of Fig. S2C. Such that the probability distribution of stoichiometries *P*(*x*) is given by:

$$P\left(x\right)=\sum_{k=1}^{5}\exp\left(-\lambda \right)\frac{\lambda^{k}}{k!}\frac{1}{\sigma \sqrt{2\pi kS}}exp\left(-\frac{x-kS}{k\sigma^{2}}\right)$$

This model is a summation of multiple Gaussian distributions which are separated by a fixed number of molecules (for example 4 molecules in the case of the tetramer model), whose amplitude scales with a Poisson distribution, as expected from the nearest neighbour model. Here *k* is the number of overlapping foci – we sum up to a maximum of *k* = 5 overlapping foci since this ensured in all cases that the expectation value of foci occurrence at higher values of *k* was<1 (i.e. *P.S* < 1 focus). Finally, each of these modelled cell stoichiometry distributions was averaged over the sporulation stage population to generate the model distribution and convolved with the same 0.7 molecule width kernel as the kernel density estimates (KDEs) [70] in the real (i.e. experimental) data. The Pearson’s Chi-squared statistic χ^2^ was calculated as:

$$\chi^{2}=\sum_{i=1}^{30}\frac{{\left(O_{i}-C_{i}\right)}^{2}}{C_{i}}$$

where the observed value *O_i_* is the normalized KDE value (i.e. scaled on the probability density axis such that the total area underneath the KDE sums exactly to 1) at single molecule bin intervals up to a total of typically *n* = 30 bins, i.e. stoichiometry range tested from the full distribution is 0–30 molecules, assuming the data contained at least one recorded focus in any respective bin (if not it was discarded in the Chi-squared summation). The calculated data value *C_i_* was taken from the normalized model fit. The degrees of freedom were equal to the number of bins used in the χ^2^ calculation subtracting the 4 free model parameters (which were overlap frequency (*λ),* max number of overlaps *(k),* intensity distribution *(*σ) and model stoichiometry (*S*)). The value of the measured χ^2^ was then used with the inbuilt inverse Chi-squared MATLAB function chi2cdf.m at this equivalent number of degrees of freedom to calculate the equivalent p value which corresponds to the null hypothesis that the measured variation between the observed values and the model fit is random. We found that the tetramer model was the only model to produce a goodness of fit corresponding to acceptable p values at approximately 0.05 or less in all stages (Fig. 3 and S3).

### 2.7 Modelling the frictional drag on SpoIIE foci

We modelled the frictional drag coefficient in the cell membrane of SpoIIE foci as that due to a cylinder whose height *h* matches the width of the phospholipid bilayer (~3nm) with a radius given by parameter *a*, using a generalized method established previously to characterize the lateral diffusion of transmembrane proteins [36], [37]. In brief, the diffusion coefficient *D* is estimated from the Stokes-Einstein relation of *D* = *k_B_T/γ*, where *k_B_* is the Boltzmann constant and *T* the absolute temperature, and the lateral viscous drag *γ* is given by:

$$\gamma =4\pi \left(\eta_{1}+\eta_{2}\right)aC\left(\varepsilon \right)$$

where *η*_1_ and *η*_2_ are the dynamic viscosity values either side of the membrane, which we assume here are approximately the same at *η*_c_ the cytoplasmic viscosity. *C* is a function of *ε* = 2*aη*_c_/*hη*_m_ where *η*_m_ is the dynamic viscosity in the membrane itself. Since *η*_m_ is typically 2–3 orders of magnitude larger than *η*_c_ [38] then *ε* is sufficiently small to use an approximation for *C* of:

$$C\approx 1/\left(\varepsilon \ln\left(2/\varepsilon \right)\right)$$

We used these formulations to generate a look-up table between *D* and *a* for the vegetative cell membrane in the mother cell, assuming *η*_m_≈600 cP, and the emerging forespore cell membrane, assuming *η*_m_≈1,000 cP, assuming *η*_c_≈1 cP throughout (Fig. 5C) [39]. We estimated a consensus value for *D* in the mother cell from the population of unweighted mean *D* values determined from all cell stages I-III (Table S2) of 1.05 ± 0.06 µ^2^m/s (±SEM, number of stages n = 5). We similarly estimated a consensus *D* value for the low mobility sporulation stages II_i_ and II_iii_ of 0.47 ± 0.04 µ^2^m/s (number of stages n = 2) and a consensus *D* value for the high mobility sporulation stages II_ii_ and III of 0.76 ± 0.05 µ^2^m/s (number of stages n = 2). We then extrapolated these consensus values and SEM error estimates using the vegetative and forespore cell membrane look-up tables to determine corresponding mean values and ± SEM ranges for *a*.

### 2.8 Stoichiometry *vs.* localization

To compare foci stoichiometry as a function of location in the forespore, a simplified, normalised 1D coordinate was used. This was based on the generous forespore segmentation which extends from the mother cell side of the septa through to the outer edge of the cell pole. There was also significant variation in the size of this segmentation between cells. Thus a normalised coordinate was used, 0–1 from the two most extreme points of the forespore. This implied that on average both the septa and cell poles lie within the most extreme points of the predicted cell outline segmentation.

### 2.9 Structural and bioinformatics analysis

CCP4mg was used to render images of structures with PDB IDs: 5MQH SpoIIE(590–827) and 5UCG SpoIIE(457–827) [40].

### 2.10 Statistics and goodness of fit

Where means are presented and compared, students t-tests were run and p-values presented. For data-driven models, such as the stoichiometry modelling, χ^2^ and p values are presented. For physical models such as FRAP and stokes fitting, the 95% confidence intervals on the fit parameters are presented as goodness of fit.

## 3 Results

### 3.1 Sporulation stage can be categorized using an accurate, high-throughput automated algorithm

We generated a chromosomally encoded fusion of SpoIIE to monomeric yellow fluorescent protein mYPet (a bright fluorescent protein with very short maturation time,<10 min [41] compared to > 2hrs sporulation time, whose long excitation wavelength results in minimal contamination of cellular autofluorescence [42]) to report on SpoIIE localization (Table S1). We prepared cells for sporulation using nutrient-depleted media, incubating with the red lipophilic dye FM4-64 for visualizing *B. subtilis* membrane structures [43]. This allowed us to observe steady-state patterns of SpoIIE-mYPet and FM4–64 localization for sporulation stages I, II (with associated sub-stages) and III with single-molecule detection sensitivity [71] via Slimfield (Fig. 1C), as well as standard epifluorescence microscopy (Fig. 1D,E and S1). We developed an automated high-throughput analysis framework using morphological transformations [33], [44] on SpoIIE-mYPet and FM4-64 data, enabling us to categorize each cell into one of five different sporulation stages (I/pre-divisional, forespore formation stages II_i_, II_ii_ and II_iii_, and III after engulfment), validated by simulation and principal component analysis (Fig. 1F,G, Fig. S1). Our algorithm segments the SpoIIE and FM4-64 images to identify septa and forespore features and categorises them into appropriate stages but does not distinguish between E-ring structures in stage I [45], [46] and SpoIIE localization in the septa in stage II_i_. The measured proportions of cells in each stage (Fig. 1G) were qualitatively similar to those reported using manual, low-throughput methods [47]. Imaging a SpoIIE-mYPet strain including a *ΔspoIIQ* deletion (Table S1), defective in spore formation and unable to progress beyond stage II_ii_, yielded similar relative proportions of cells in stages I, II_i_ and II_ii_ (Fig. S1). Although imperfect, resulting in some mis-characterisation (Fig. S1), our software is objective and enables study of cells which are not easily categorised by eye and avoids biasing our study to just previously accepted morphological features of sporulation.

### 3.2 SpoIIE is concentrated in the forespore, probably through equipartitioning

**Fig. 2:**
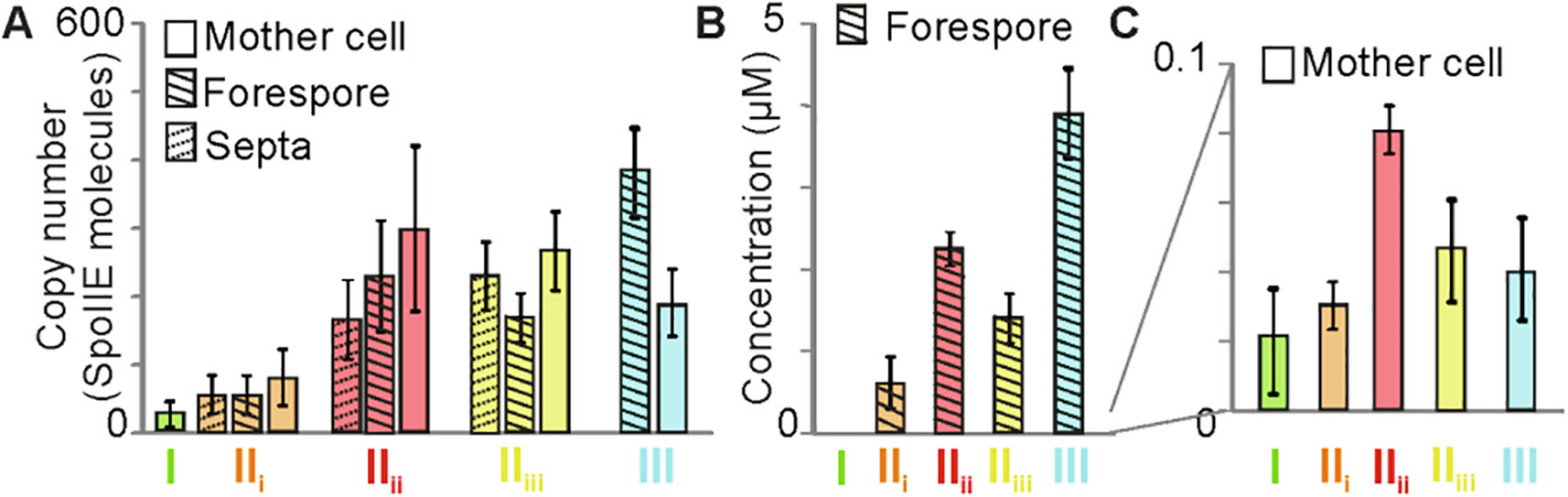
. SpoIIE copy number (A) Mean and SEM SpoIIE copy number in mother cell, forespore and septa at each stage. (B) Concentration in the forespore, excluding the septa, at each stage. (C) Concentration in the mother cell, excluding the septa at each stage. Stage I (green),II_i_ (orange), II_ii_ (red), II_iii_ (yellow) and III (cyan). Mother cell copy numbers statistically higher than stage I by stage II_ii_ (p = 0.02) and forespore copy number increases significantly between stage II_i_ and II_ii_ (p = 0.007). (For interpretation of the references to color in this figure legend, the reader is referred to the web version of this article.)

Slimfield images revealed distinct foci, as well as a more diffuse pool of fluorescent SpoIIE localized close to the cell membrane as expected (Movies S1 to S3). Slimfield employs a high numerical aperture objective lens with a high depth of field, thus a significant amount of fluorescence and even foci were detected in the middle of the cell. To check that this signal was really from membrane bound SpoIIE, we simulated images of membrane bound fluorophores in model *Bacillus* shaped cells and found similar patterns of localization (Fig. S1B). We used bespoke single particle localization [48] on the Slimfield data to track foci whose width was consistent with the measured point spread function (PSF) of our microscope, ~250 nm. Foci could be tracked over consecutive images up to ~ 0.3 s using rapid 5 ms per frame sampling to a spatial precision of 40 nm [27]. Tracking of distinct foci was coupled to molecular stoichiometry analysis by estimating the initial foci brightness and dividing this by the brightness of a single mYPet molecule [22], [24], [31], [49], [50] (Fig. S2A-C). We also observed a more diffuse pool of mYPet fluorescence, not detected as foci or caused by cell autofluorescence which was negligible. Slimfield images were taken at the approximate cell mid-body so foci at the top or bottom of the cell membrane are outside the depth of field, generating the more diffuse fluorescence observed. By using integrated pixel intensities [27], we determined the total SpoIIE copy number for each cell. Utilizing our stage categorization algorithm we assigned each cell to one of the sporulation stages I-III, and also sub-divided each into three sub-regions – a septum contributed by both mother cell and forespore, a mother cell which excluded the septum, and a forespore which excluded the septum. We then quantified the number of SpoIIE molecules specifically associated with each of these sub-regions for each cell imaged (Fig. 2A, S2D).

These analyses (Table S2) show that the total SpoIIE copy number starts at a few tens of molecules per cell in stage I, increasing as sporulation progresses to ~ 200 SpoIIE in stage II_i_, then rising to 700–800 molecules per cell in stages II_ii_ and II_iii_, before dropping down to ~ 580 molecules per cell in stage III after spore engulfment. The mother cell sub-region excluding the septum reflects this trend, increasing SpoIIE copy number from 20 to 80 molecules between stages I-II_i_, peaking at ~ 300 molecules in stages II_ii_-II_iii_, then tailing off to ~ 190 molecules in stage III. Copy number in the septum and forespore also increases throughout sporulation, starting at ~ 60 copies of SpoIIE at stage II_i_ increasing to ~ 400 copies by stage III.

A key question is whether the SpoIIE concentration is higher in the forespore than the mother cell, providing an explanation for cell-specific σ^F^ activation [19]. Our results support this hypothesis, although they are complicated by ambiguity in septal fluorescence, which has potential contributions from both the mother cell and the forespore, since the standard optical resolution limit is greater than the pixel-level precision of image segmentation algorithms. Even excluding the septal region, the forespore concentration of SpoIIE is an order of magnitude higher than that in the mother cell (Fig. 2B and C). This increased concentration would arise from equipartitioning of SpoIIE between the mother cell and forespore combined with the ~ 6 times smaller volume of the forespore. We use volume as the simplest model here but similar results are obtained using the ~ 3 times smaller surface area to also account for SpoIIE being membrane bound. Intriguingly, arbitrarily attributing the septal fluorescence equally to the mother cell and forespore, the simplest model considering the ambiguity in which side it is on, results in approximately equal copy numbers in the two. It has been shown with an in vitro reconstituted system that a ~ 10-fold increase in the phosphatase activity of SpoIIE towards SpoIIAA ~ P is sufficient to release 90% of σ^F^ from its inhibitory complex[51]. However, this imbalance in SpoIIE concentration cannot be immediately decisive *in vivo* as σ^F^ activation is delayed until stage II_ii_. This suggests that following septation either SpoIIE is not immediately active as a phosphatase, or that following its dephosphorylation by SpoIIE, SpoIIAA is delayed in its capacity to displace σ^F^ from its inhibitory complex with the anti-sigma factor.

### 3.3 SpoIIE is a tetramer whose quaternary organization depends on spatial and temporal localization

**Fig. 3:**
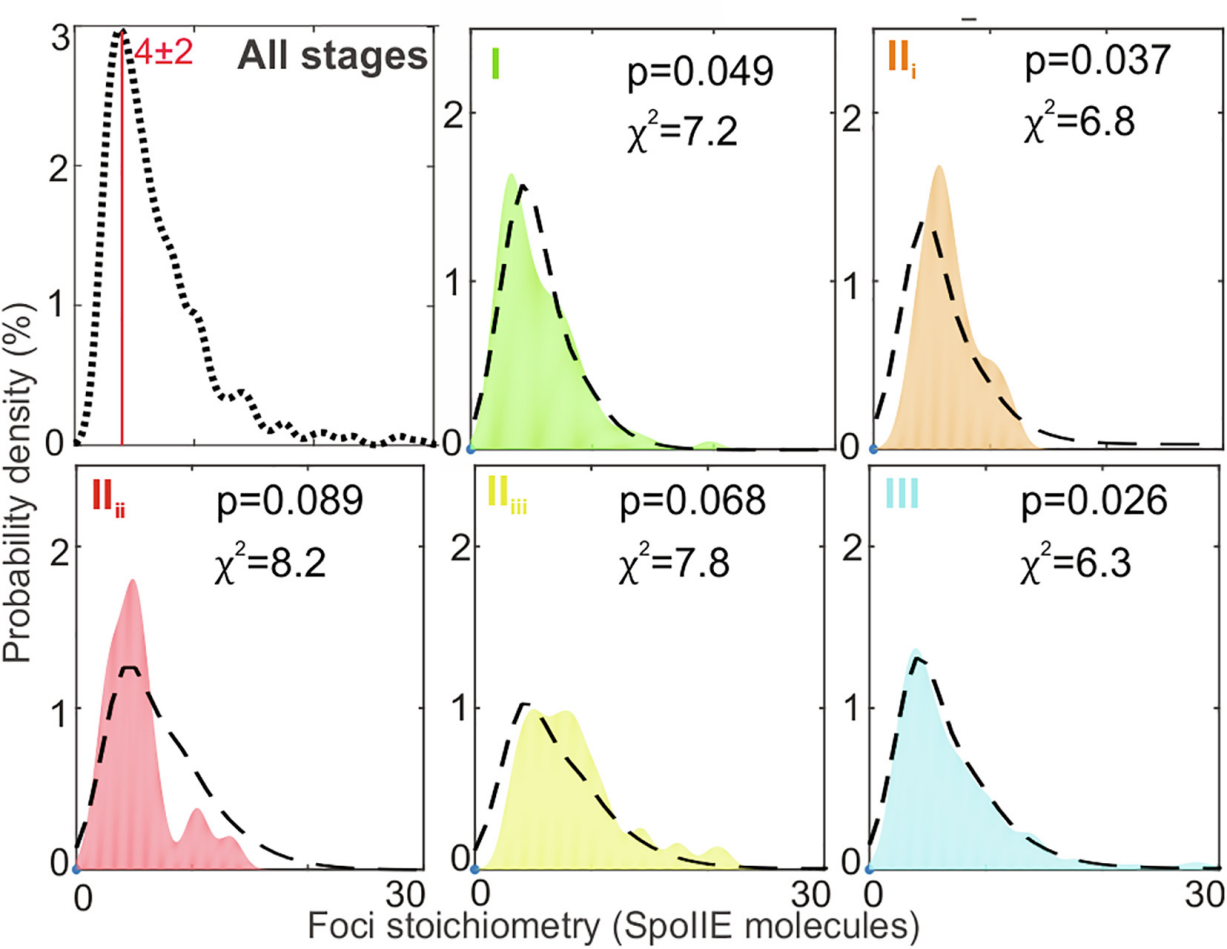
. Stoichiometry of SpoIIE in the mother cell. Kernel density estimation (KDE) of the stoichiometry (i.e. number of SpoIIE molecules per detected fluorescent focus) in mother cell with predicted overlap tetramer model (black dotted line).Chi-squared χ^2^ and probability of confidence p values indicated. Stage I (green),II_i_ (orange), II_ii_ (red), II_iii_ (yellow) and III (cyan). (For interpretation of the references to color in this figure legend, the reader is referred to the web version of this article.)

Next, we sought to characterize the molecular architecture of functional SpoIIE by measuring the stoichiometry of fluorescent foci. In the mother cell, the apparent stoichiometry of tracked foci ranged from as few as two up to several tens of molecules, but with a clear peak at 4 ± 2 SpoIIE molecules, conserved throughout stages I-III (Fig. 3). Using a randomized Poisson model for nearest-neighbour foci distances, whose key parameters comprise SpoIIE copy number and foci density, we calculated the probability of foci being separated by less than the optical resolution limit (thus detected as single foci of higher apparent stoichiometry) to be 20–40% in the mother cell. Overlap models which used the raw SpoIIE-mYPet intensity distribution (Fig S2C) in monomers, dimers, hexamers or octamers do not account for the observed stoichiometry distribution (Fig. S3). By contrast, we find that a tetramer overlap model generates reasonable agreement within experimental error for stages I-III in the mother cell (Fig. 3, dashed lines) for all stages, with a corresponding mean probability of confidence value of p = 0.05. Thus, we believe the most likely model among those trialled is that SpoIIE in the mother cell comprises predominantly tetramers.

**Fig. 4:**
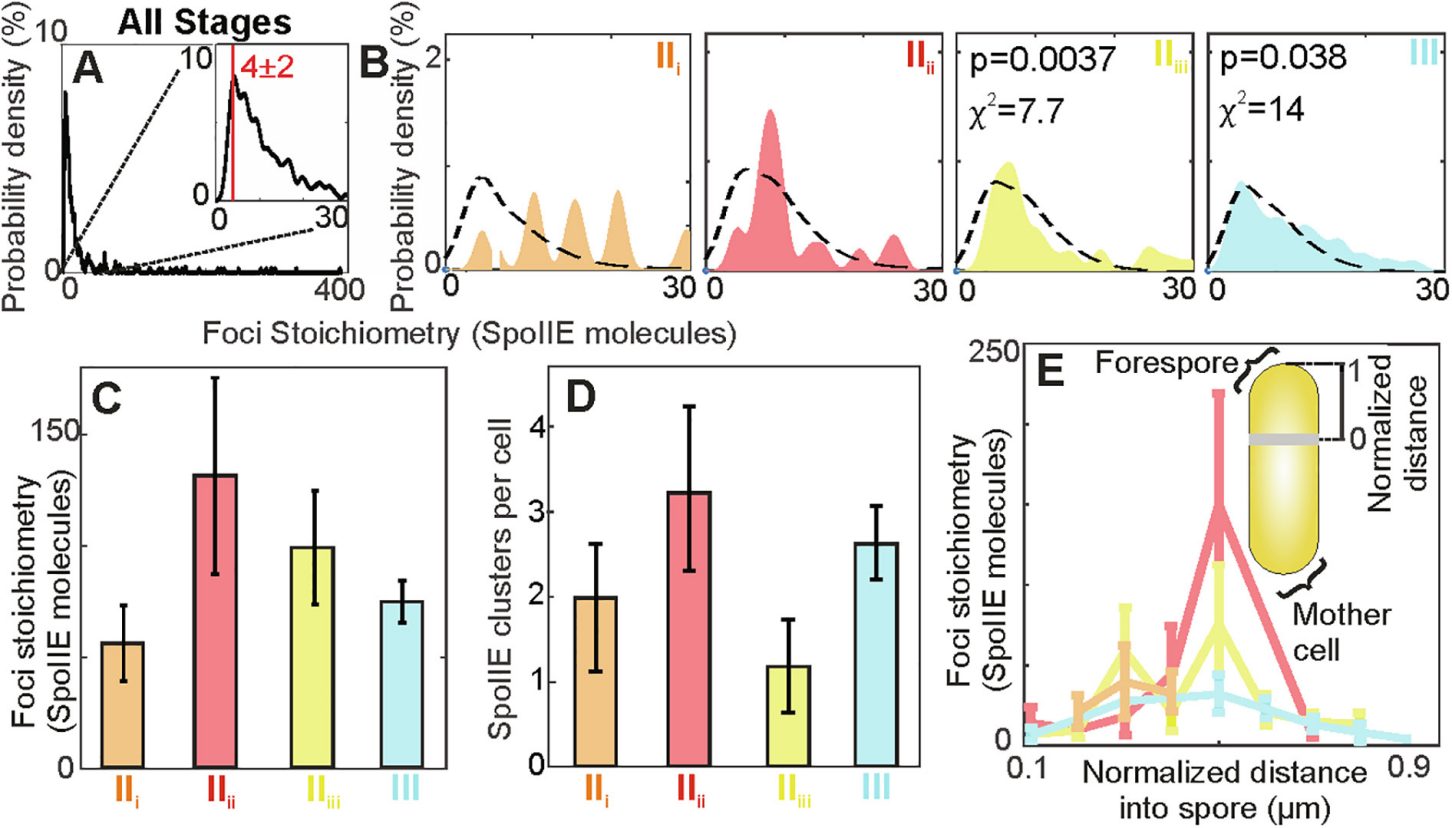
. Stoichiometry of SpoIIE in the forespore. (A) Kernel density estimate (KDE) of forespore stoichiometry pooled for all data, and zoom-in (inset), (B) in separate stages with overlap tetramer model (black dotted line). (C) Bar chart of mean foci stoichiometry for foci which have > 20 molecules. Stage II_ii_ statistically higher than II_i_ (p = 0.007) (D) Mean number of foci detected per forespore. (E) Mean foci stoichiometry *vs.* normalised distance into spore for each stage, (inset) schematic of forespore distance normalization. Stage I (green),II_i_ (orange), II_ii_ (red), II_iii_ (yellow) and III (cyan). (For interpretation of the references to color in this figure legend, the reader is referred to the web version of this article.)

For SpoIIE foci in the forespore or the septum, we find the same tetramer peak in the measured stoichiometry distribution but with a longer tail of higher stoichiometry clusters extending up to hundreds of SpoIIE molecules per focus (Fig. 4A, B). We adapted the overlap model to account for different sizes and shapes of sporulation features at each stage resulting in differences in the density of SpoIIE foci (Fig. 4B). The overlapping tetramer model accounts only for low apparent stoichiometries near the tetramer peak and only in stages II_iii_ and III. More generally, accounting for the apparent stoichiometry in the forespore requires populations of higher order oligomeric SpoIIE clusters in the model fit, in addition to tetramers. Excluding free tetrameric foci, we observe 1–3 clusters per cell (Fig. 4D) with the mean cluster stoichiometry peaking in stage II_ii_ at > 100 molecules per focus (Fig. 4C) before decreasing as the proportion of free tetramers increases again in stage III (Fig, S5A, B). We find for foci present in the forespore the measured stoichiometry in all stages was periodic, with a characteristic interval spacing of ~ 4 molecules (Fig. S4), suggesting that higher order clusters are composed of associating SpoIIE tetramers.

Aspects of these *in vivo* observations are consistent with previous *in vitro* experiments. Analytical ultracentrifugation experiments using a soluble fragment of SpoIIE, in which the N-terminal 319 residues, which includes the 10 putative transmembrane segments, were truncated, suggested that SpoIIE(319–872) formed hexamers and larger assemblies composed of multiples of hexamers. [20] A more recent study of a similarly truncated protein SpoIIE(325–872) fragment fused to maltose binding protein demonstrated reversible manganese-dependent oligomerisation as evidenced by changes in sedimentation behaviour and the observation of extended structures (50 nm × 10 nm) using electron microscopy [52], although these authors did not speculate on the oligomeric state of the species involved. Fragments of SpoIIE are challenging to express and purify (see also Lucet et al., 2000 [12]) and their behaviour is sensitive to the size of the truncation. It is therefore not surprising that the full length protein present in the membranes of living cells assembles in a different manner. Whether SpoIIE forms oligomers *in vivo* in the absence of manganese would be an interesting topic of further study.

We also observed that the stoichiometry of foci in the forespore was influenced by their distance from the septum. We normalized the distance parallel to the long axis of each cell from the mother cell side of the asymmetric septum through to the distal outer edge of the cell containing the smaller forespore cell for all tracked foci and plotted this distance against foci stoichiometry (Fig. 4E and S5C)., For stage II_i_, foci are localized to the septum, within ~ 300 nm , however, other stages contain foci which are delocalized over the full extent of the emerging forespore (Fig. 4E); we find that the mean SpoIIE stoichiometry for these foci increases from ~ 12 to 150 molecules per focus (a factor of ~ 12) for stage II_ii_. This observation supports the recently proposed mechanism for σ^F^ activation regulation [20] through clustering of SpoIIE in the direction of the pole at stage II_ii_

### 3.4 SpoIIE foci mobility suggests that large multi-protein assemblies are present in stages II_i_ and II_iii_

**Fig. 5:**
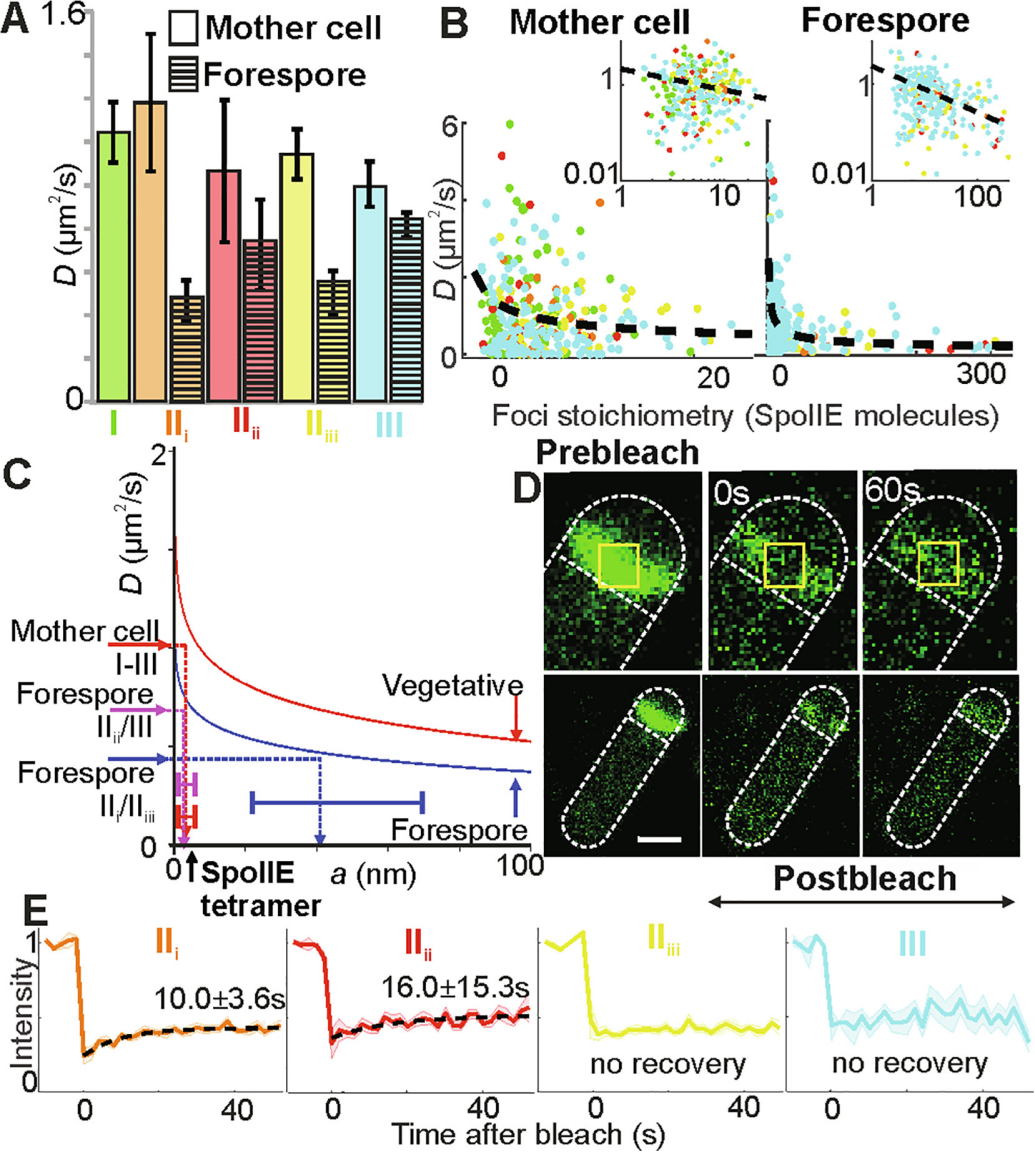
. SpoIIE mobility (A) Bar chart of mean *D* for each compartment and stage, SEM errors. Forespore statistically different to mother cell in stages II_i_ and II_iii_ (p = 0.018, 0.284, 0.004, 0.160, respectively for each stage. No significant change between each stage in the forespore , except III, p = 0.003). (B) Scatter plots of stoichiometry *vs. D* for all stages in mother cell and forespore, log–log axes inset (power law model black line). (C) Variation of *D* with effective cluster cylinder radius *a* from frictional drag model, vegetative (i.e. as opposed to sporulating, shown in red) and forespore (blue) indicated with interpolations from *D* to *a* made from mother cell (all stages), and forespore stages (II_i_/II_iii_) and (II_ii_/III), SEM errors. (D) FRAP from representative cell in stage II_i_, immediately pre/post-bleach, and ~ 60 s post-bleach, bleached region indicated (yellow squares). (E) Mean normalised fluorescence recovery for each stage, SEM bounds shown (shading). Dotted lines show best exponential fit where recovery was detected, *t_r_* and 95% confidence intervals indicated. Stage I (green),II_i_ (orange), II_ii_ (red), II_iii_ (yellow) and III (cyan). N = 10–20 cells per stage. (For interpretation of the references to color in this figure legend, the reader is referred to the web version of this article.)

We sought to determine the composition and function of clusters by analyzing their mobility in live cells. We find that SpoIIE fluorescent foci mobility in general was consistent with Brownian (i.e. normal) diffusion over short timescales irrespective of cell compartment or stage (Fig. S6, S7). In the mother cell, the mean value of the microscopic diffusion coefficient *D* was 0.9–1.2 μm^2^/s while that in the forespore was lower by a factor of ~ 2 (Fig. 5A, Fig. S7 and Table S2). At the onset of sporulation in stage II_i_ foci mobility in the forespore is at its lowest with a mean *D* of 0.43 ± 0.08 μm^2^/s, which increases during stage II_ii_ to 0.67 ± 0.19 μm^2^/s, then decreases in stage II_iii_ to 0.50 ± 0.09 μm^2^/s before increasing again in stage III to 0.76 ± 0.05 μm^2^/s, although only statistically significant in stage III. For stages I-III, *D* shows a dependence on stoichiometry *S*, indicating a trend for decreasing *D* with increasing SpoIIE content (Fig. 5B). Modelling this dependence as *D* ~ *S^α^* indicates a power-law exponent *α* of 0.48 ± 0.18, with no measurable difference within error for each stage (Fig. S7).

Calculations of frictional drag on SpoIIE foci, using a consensus value for *D* from stages I-III for the mother cell, indicate an average Stokes radius (the radius of equivalent cylinder in the membrane) in the range 3–8 nm (Fig. 5C, red dashed line). The N-terminal 330 residues of SpoIIE are predicted to form a membrane binding domain with 10 transmembrane α-helices [53]. A close packed circular arrangement of these helices, each with a diameter of 1.2 nm, would produce a SpoIIE tetramer comprising 40 transmembrane helices with a ~ 4 nm radius, consistent with our experimentally-derived estimate. By contrast, a ‘mean’ ~50-mer SpoIIE cluster has a Stokes radius of ~ 13 nm. Thus the Stokes radius provides an estimate for the real size of the diffusing SpoIIE complex, including any other protein partners diffusing along with it.

For the forespore, the mean value *D* for higher SpoIIE mobility stages II_ii_ and III indicates a range for Stokes radius consistent with clusters composed solely of SpoIIE tetramers (Fig. 5C, magenta dashed line). However, the low SpoIIE mobility stages II_i_ and II_iii_ indicate a Stokes radius approximately an order of magnitude higher at ~ 40 nm (Fig. 5C, blue dashed line), far more than expected for a cluster of only 100 SpoIIE molecules. This observation supports a model in which SpoIIE interacts with other proteins or complexes, with these other unlabeled proteins here forming ~ 5x the SpoIIE foci surface area in the membrane, increasing the apparent Stoke’s radius. In stage II_i_ interactions would be with components of the divisome [12], [13], [14] while in stage II_iii_ they would be with SpoIIQ, the forespore component of an intercellular channel formed with proteins encoded on the *spoIIIA* operon expressed in the mother cell [15]. In stage II_ii_ we find clusters of SpoIIE are likely not associated with a protein partner as the Stokes radius is consistent only with the SpoIIE present. This finding is also consistent with σ^F^ activation regulation [20].

### 3.5 Forespore SpoIIE turnover depends on sporulation stage

Using confocal microscopy of a similar cell strain but using monomeric GFP labelled SpoIIE (i.e. SpoIIE-mGFP) we performed fluorescence recovery after photobleaching (FRAP) experiments to photobleach the asymmetric septum at different stages and monitor any subsequent fluorescence recovery (Fig. 5D). During stages II_i_ and II_ii_ there is a relatively slow recovery with mean exponential recovery time *t_r_* of 10 ± 3.6 s and 16.0 ± 15.3 s respectively (Fig. 5E, S7). Our finding that *t_r_* is not directly correlated to *D* in each stage suggests that turnover here is reaction- as opposed to diffusion-limited; it may be limited by an effective off-rate as observed in other complex bacterial structures such as components of the flagellar motor or replisome [54], [24]. In subsequent stages II_iii_ and III, no recovery is detectable within error, though lower levels of fluorescence and numbers of cells in stage III result in higher measurement noise which limits the sensitivity for detecting low levels of putative recovery.

Divisome components such as FtsZ have been shown to turnover in similar FRAP studies [55], consistent with our stage II_i_ findings when SpoIIE associates with the divisome. Turnover is also expected at stage II_ii_ when SpoIIE is released. At stage II_iii_ SpoIIE interacts with the SpoIIQ-SpoIIIAH channel which may account for the lack of turnover. A similar absence is unexpected at stage III when SpoIIE is released and has no known function. This suggests that at stage III, SpoIIE is released quickly then anchored into the spore, or that the viscosity in spore itself has changed as has been shown to occur during sporulation [39].

## 4 Discussion

SpoIIE performs multiple important functions. For example, it is essential to form a proper sporulation septum as well as to activate SigF. Without SpoIIE no spore can be formed and also there are many point mutations characterized in *spoIIE* which cause complete arrest of cell differentiation. However, how SpoIIE switches roles at different stages has been unclear. It is not known how SpoIIE localizes to the polar septum, how it causes FtsZ to relocalize from mid-cell to one of the cell poles, what role it plays in septal thinning, or how its SpoIIAA-P phosphatase activity is controlled so that σ^F^ activation is delayed until the asymmetric septum is completed [7], [11]. How SpoIIE brings about forespore-specific activation of σ^F^ is a subject of particular interest [56]. Plausible suggested mechanisms include preferential SpoIIE localization on the forespore face of the septum [57], transient gene asymmetry leading to accumulation of a SpoIIE inhibitor in the mother cell [56], and the volume difference between compartments leading to higher specific activity of equipartitioned SpoIIE [58], [59]. Most recently, it was shown that mother cell restricted intracellular proteolysis of SpoIIE by the membrane bound protease FtsH is important for compartment-specific activation of σ^F^ [20]. Our findings indicate that SpoIIE operates as an oligomer whose stoichiometry and mobility switch in the forespore according to specific sporulation stage, driving morphological changes, as opposed to changes being primarily dependent on the differential effective concentration of SpoIIE in either mother cell or forespore. In particular, complexes comprising four SpoIIE molecules predominate in the mother cell and at multiple stages in the forespore. Crucially, we observe reversible assembly of these tetrameric SpoIIE entities into higher order multimers during stage II_ii_ when the protein localizes towards the pole and its latent protein phosphatase activity is manifested.

Unlike previous microscopy of YFP-labelled SpoIIE which suggested a pattern of localization almost exclusively in the forespore following asymmetric septation [20], our higher sensitivity shows SpoIIE content is at most 10–30% greater in the early forespore and septum compared to the mother cell if all of the SpoIIE in the septum is assigned to the forespore. An equipartition of septal SpoIIE results in approximately equal copy number in the mother cell and forespore. However, the >6 times smaller forespore volume [19] results in a higher SpoIIE concentration by a factor of ~ 6–8, depending on partitioning of septal SpoIIE. It was shown previously that a 10-fold difference in SpoIIE phosphatase activity towards its substrate SpoIIAA ~ P could account for all-or-nothing compartmental regulation of σ^F^ activity [60]. The bias towards higher copy number values in the forespore aligns with the recent suggestion that SpoIIE captured at the forespore pole is protected against proteolysis [20]. In this model, SpoIIE sequestered in the polar divisome, is handed-off to the adjacent forespore pole following cytokinesis. This forespore polar SpoIIE is protected from FtsH-mediated proteolysis by oligomerisation, which is clearly described by our observations. Compartment specificity results from the proximity of the forespore pole to the site of asymmetric division.

**Fig. 6:**
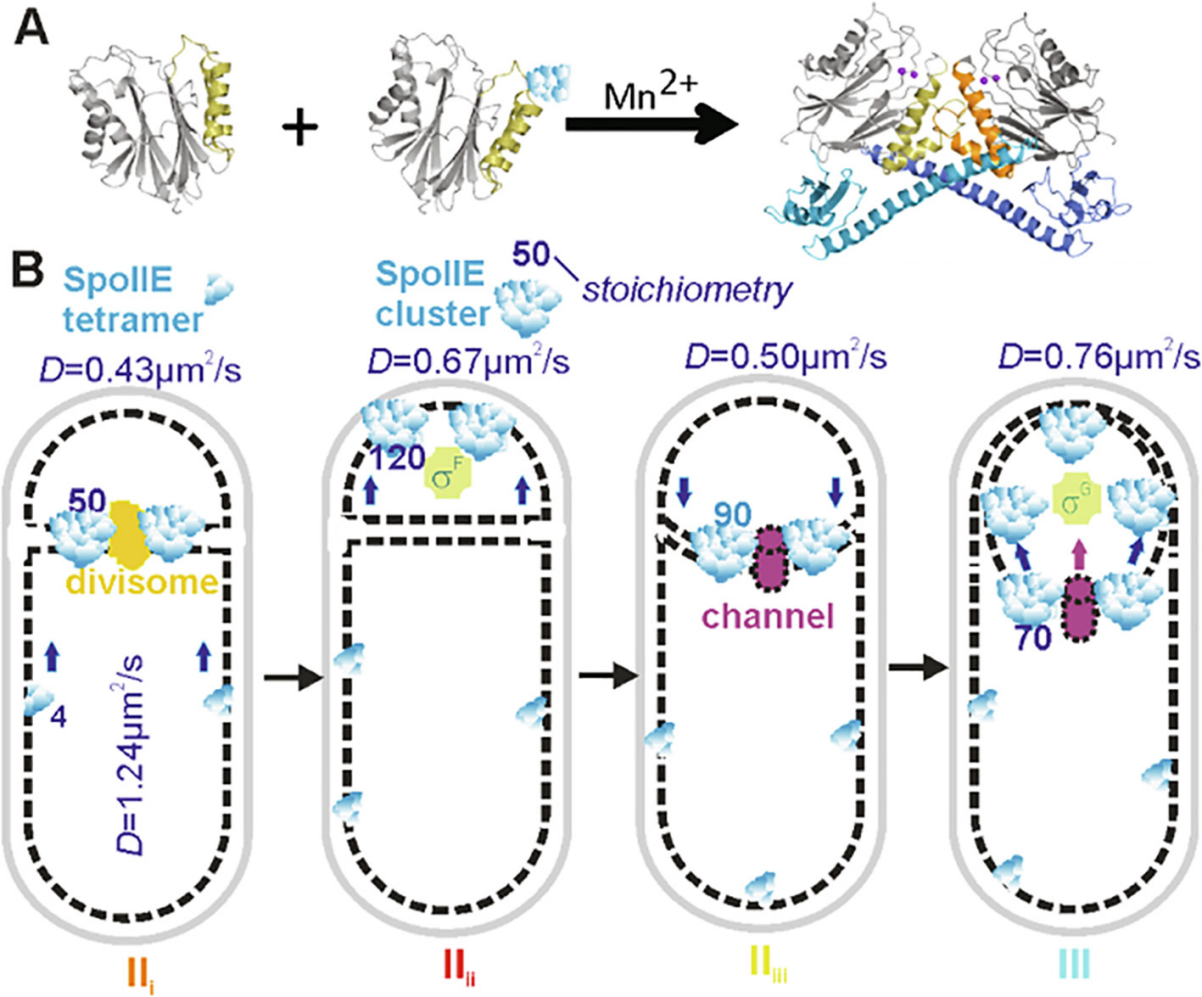
. Model of SpoIIE dynamics during sporulation. (A) Activation of phosphatase involves dimerization, and recruitment of Mn^2+^ ions. Isolated phosphatase, SpoIIE(590–827) (PDB: 5MQH), is monomeric while a longer fragment, SpoIIE(457–827) (PDB: 5UCG), forms dimers about an interface dominated by a long helix spanning residues 473–518. Subunits in dimer distinguished by shading: PP2C domains (gray), switch helices (orange), regulatory domains (blue), Mn^2+^ ions (purple spheres, modeled onto structure following superposition of PstP structure from *M. tuberculosis* PDB: 1TXO). (B) Schematic of SpoIIE architecture and *D* at each stage: SpoIIE tetramers (blue), divisome (yellow), activated sigma factors (green), and SpoIIQ channel (purple) shown. Arrows indicate SpoIIE’s release or re-capture to the septa. (For interpretation of the references to color in this figure legend, the reader is referred to the web version of this article.)

Crystallographic and biophysical studies reveal that SpoIIE(590–827), comprising the phosphatase domain, is a monomer while SpoIIE(457–827), comprising the phosphatase plus part of the upstream regulatory domain, is dimeric (Fig. 6A)[61], [62]. Comparison of these structures and mapping of mutational data onto them led to the proposal that PP2C domains in SpoIIE(590–827) and SpoIIE(457–827) crystals represent inactive and active states respectively. Activation is accompanied by a 45° rigid-body rotation of two ‘switch’ helices [62]. This switch is set by a long α-helix in the regulatory domain which mediates dimerization (Fig. 6A). Movement of the switch helices upon dimerization translates a conserved glycine (Gly629 in SpoIIE) into the active site where it can participate in cooperative binding to two catalytic manganese ions. These ions are conserved in PP2C phosphatases and here would be expected to activate a water molecule for nucleophilic attack at the phosphorus of the phosphorylated serine 57 residue in SpoIIAA-P. The increase in SpoIIE stoichiometry observed upon activation *in vivo* is consistent with these structural findings although clearly larger assemblies are implied. We can speculate on the basis of the data presented here that these larger assemblies arise from further homomeric quaternary interactions mediated by the substantial membrane binding domain and/or the component of the regulatory domain which has yet to be fully characterized. The results are consistent with the hand-off model [20] in which release from the divisome allows SpoIIE tetramers to diffuse away from the septum and self-associate to form high stoichiometry clusters in a spontaneous process with similarities to that observed for the plasmalemmal protein syntaxin[63]. We speculate that the free energy of reassembly is used to flip the helical switch, allowing manganese acquisition and activation of phosphatase activity.

Changes in oligomeric state and quaternary organization of proteins are widespread mechanisms for regulating biological activity. These can be induced variously by binding of allosteric ligands, covalent modification, proteolytic processing and reversible interactions with protein agonists or antagonists. SpoIIE, which transitions between complexes which are unusually large, is transiently active as a phosphatase after its release from an inhibitory complex with the divisome. Regulation of phosphatase activity through sequestration is also seen in adaptation to drought in plants; the phosphatase HAB1 dephosphorylates the kinase SnRK2, inhibiting transcription of drought tolerance genes until the complex of the hormone, abscisic acid and its receptor, PYR, binds to and inhibits the phosphatase HAB1 [64].

Our findings show more generally that we can combine robust cell categorization with single-molecule microscopy and quantitative copy number and stoichiometry analysis to follow complex morphologies during differentiation (Fig. 6B). Importantly, these tools provide new insight into the role of SpoIIE by monitoring its molecular composition and spatiotemporal dynamics, linking together different stages of cell development. Our findings show that the function of a key regulatory protein can be altered depending upon its state of multimerization and mobility, enabling different roles at different cell stages. Future applications of these methods may involve multicolor observations of SpoIIE with other interaction partners at different sporulation stages. Optimising these advanced imaging tools in the model Gram-positive *B. subtilis* may ultimately enable real time observations of more complex cellular development, paving the way for future studies of tissue morphogenesis in more challenging multicellular organisms. More generally our findings demonstrate that the application of super-resolved single-molecule optical proteomics biotechnology can enable new mechanistic insight into complex cell stage dependent processes in single living cells which are technically too challenging to achieve using traditional methods [72], [73], [74], [75]. Such findings are made possible by a range of innovative computational tools to categorise cell cycle stage and to quantify single-particle tracks, and enable not only new understanding of the dynamic patterns of spatial localization of a key protein used in triggering cell development, but also in posing questions about its structural properties at different cell cycle stages.

## 5 Data availability

Data included in full in main text and supplementary files. Raw data available from authors.

## 6 Software access

Code written in MATLAB available from Sporulationanalyser at https://sourceforge.net/projects/york-biophysics/

## Author contributions

I.B., A.J.W. and M.C.L. designed research; A.J.M.W, K.M. and Z.C. performed research; A.J.M.W., K.M. and Z.C. analyzed data; A.J.M.W., I.B., A.J.W. and M.C.L. wrote the paper.

## Declaration of Competing Interest

The authors declare that they have no known competing financial interests or personal relationships that could have appeared to influence the work reported in this paper.
